# Routine MRCP in the management of patients with gallbladder stones awaiting cholecystectomy: a single-centre experience

**DOI:** 10.1007/s13244-018-0640-3

**Published:** 2018-07-05

**Authors:** Valentina Virzì, Noemi Maria Giovanna Ognibene, Antonio Salvatore Sciortino, Glenda Culmone, Giuseppe Virzì

**Affiliations:** 1 Department of Radiology, “Regina Pacis” Clinic, via Principe Lanza di Scalea 3/5, 93017 San Cataldo, CL Italy; 2 Department of Surgery, “Regina Pacis” Clinic, via Principe Lanza di Scalea 3/5, 93017 San Cataldo, CL Italy; 3 “Regina Pacis” Clinic, Skema Iniziative Sanitarie, via Principe Lanza di Scalea 3/5, 93017 San Cataldo, CL Italy

**Keywords:** Gallbladder stones, MRCP, Choledocholithiasis, MRI, US

## Abstract

**Objectives:**

To assess the frequency of choledocolithiasis and the role of preoperative laboratory findings, ultrasound (US) and magnetic resonance cholangio-pancreatography (MRCP) in the detection of choledocolithiasis in patients with gallbladder stones awaiting cholecystectomy.

**Methods:**

A consecutive sample of 104 patients underwent MRCP prior to cholecystectomy. The patients were classified into different groups on the basis of the risk of choledocolithiasis. A specialised doctor with more 10 years of experience performed the US interpretation and a radiologist performed the MRCP interpretation blinded to US or aspartate aminotransferase (AST)/alanine aminotransferase (ALT)/alkaline phosphatase (ALP) results. A chi-square (χ^2^) test was performed to assess the statistical significance of differences in the frequency of choledocolithiasis based on laboratory findings, choledocal diameter on US and group risk.

**Results:**

MRCP showed calculi in 7 out of 104 patients (6.7%), with no statistically significant differences between the high/moderate risk and low/no risk groups and between the patients with normal and altered laboratory findings or choledocal diameter on preoperative US. The sensitivity and specificity of AST/ALT [positive predictive value (PPV): 12%; negative predictive value (NPV): 94%], ALP (PPV: 7%; NPV: 94%), total serum bilirubin (PPV: 6%; NPV: 93%) and choledocal diameter (PPV: 20%; NPV: 94%) were, respectively, 28.6 and 94.8%, 85.7 and 17.5%, 14.3 and 93.8%, and 14.3 and 95.9%.

**Conclusions:**

MRCP is a reliable evaluation for the detection of common bile duct (CBD) stones, reducing the misdiagnosis of retained choledocholithiasis with normal biochemical predictors and US examination.

**Main messages:**

*• MRCP is a non-invasive method for the detection of CBD stones.*

*• Preoperative MRCP reduces the misdiagnosis of retained choledocholithiasis.*

*• Detection of choledocholithiasis is mandatory prior to cholecystectomy to avoid surgical morbidity*

## Introduction

Common bile duct (CBD) stones may occur in up to 5–15% of patients with symptomatic gallstone disease [1]. Many clinical algorithms, based on clinical, biochemical and radiological indices, have been well coded in order to assess the utility of preoperative assessment of the CBD [1–5]. Choledocholithiasis may be asymptomatic but it increases the risk of development of complications, with major morbidity and mortality, so the detection and treatment of CBD stones is mandatory [2].

Magnetic resonance cholangio-pancreatography (MRCP) is a reliable tool to study the biliary tree, with a diagnostic accuracy of almost 100% in demonstrating CBD stones [2], and previous studies assessed the role of MRCP in selecting patients with CBD stones for preoperative endoscopic sphincterotomy [3, 4]. Other studies evaluated the role of liver function tests and ultrasound (US) in predicting CBD lithiasis [6, 7].

We collected preoperative laboratory findings and investigated routinely our patients through MRCP and US prior to cholecystectomy, in order to answer the following questions: what is the predictive value of liver function tests and morphological features on abdominal US for CBD stones? What is the frequency of CBD stones? Is it possible to identify patients at risk of choledocholithiasis? Is it useful to perform preoperative MRCP in all patients before cholecystectomy?

## Materials and methods

### Patients

We prospectively collected the data of patients who underwent cholecystectomy because of a gallstone disease between January 2012 and December 2013 at the “Regina Pacis” Clinic, San Cataldo (CL), Italy. Data collected included clinical characteristics, preoperative serum total bilirubin, alkaline phosphatase (ALP), alanine aminotransferase (ALT), aspartate aminotransferase (AST) and choledocal diameter on preoperative US.

Among 149 patients, 45 could not undergo MRCP because of claustrophobia, certain pacemakers, implantable cardiac defibrillators or deployable metallic prostheses. Thus, only 104 patients (75 female; mean age 53.5 years, range 17–83) were included in the study and underwent preoperative unenhanced MRCP (1.5 Tesla, Toshiba Vantage Titan scanner).

If a CBD lithiasis was found, preoperative extraction by endoscopic retrograde cholangio-pancreatography (ERCP) was attempted; then, laparoscopic cholecystectomies were performed by two surgeons.

The details of all patients were entered into a spreadsheet (Excel).

We divided the patients into different risk groups as suggested by Kim et al. in 2002 [8]: high, moderate and low risk (see Table 1), according to established criteria, including clinical characteristics (presence of cholangitis), biochemical abnormalities (serum bilirubin, ALT, AST, ALP) or morphological features on abdominal US (CBD dilated more or less than 8 mm). All the patients not included in these three groups were considered as having no risk for CBD lithiasis.

**Table 1:** Classification of clinical risk group according to laboratory and sonographic findings [8]

Risk group	Findings
High	- Presence of jaundice or cholangitis
	- Acute biliary pancreatitis
	- Total bilirubin > 1.5 mg/dL
	- ALP > 220 U/L
	- CBD stone suspected but not diagnostic at sonography
	- CBD diameter at sonography > 8 mm
Moderate	- Total bilirubin > 1.2 and < 1.5 mg/dL
	- ALP > 110 and < 220 U/L
	- Status of posibiliary pancreatitis
	- AST or ALT > 100 U/L
Low	- Atypical abdominal pain or biliary colic
	- Previous jaundice
	- Elevation of AST or ALT ≤ 100 U/L

The study was approved by the institutional ethics committee.

### Statistical analysis

The statistical analysis was performed by using IBM SPSS Statistics 20.0.

A chi-square (χ^2^) test was performed to assess the statistical significance of differences in the frequency of CBD lithiasis based on laboratory findings, choledocal diameter on US and group risk according to Kim et al.’s classification. A *p*-value < 0.05 (χ^2^ > 3.84) was considered significant. Moreover, we assessed the positive and negative predictive values (PPVs and NPVs, respectively) of biochemical abnormalities and morphological features on abdominal US.

## Results

On clinical examination, most of the patients complained of upper abdominal pain or biliary colic, which were present in 93 out of 104 patients (89.4%), but none of them demonstrated cholangitis.

Regarding the preoperative laboratory findings, 17 patients had elevated AST (range 42–275 IU/L) and ALT (range 51–382 IU/L), 86 patients had elevated ALP (range 112–900 IU/L) and 18 patients had elevated total serum bilirubin (range 1.3–6.6 mg/dL).

**Fig. 1:**
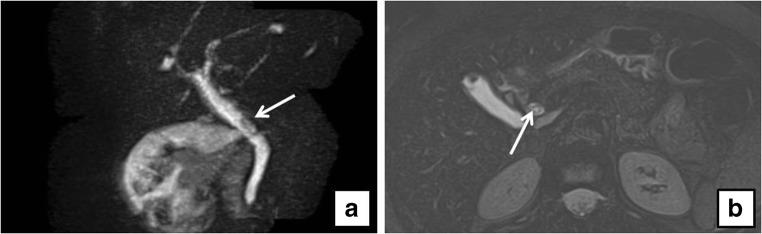
**a** Sagittal thick-slab magnetic resonance cholangio-pancreatography (MRCP) shows a filling defect in a dilated common bile duct (CBD) (*arrow*). **b** Axial fat-saturated T2-weighted magnetic resonance imaging (MRI) of the same patient shows a dishomogeneous filling defect in a dilated CBD (*arrow*)

**Fig. 2:**
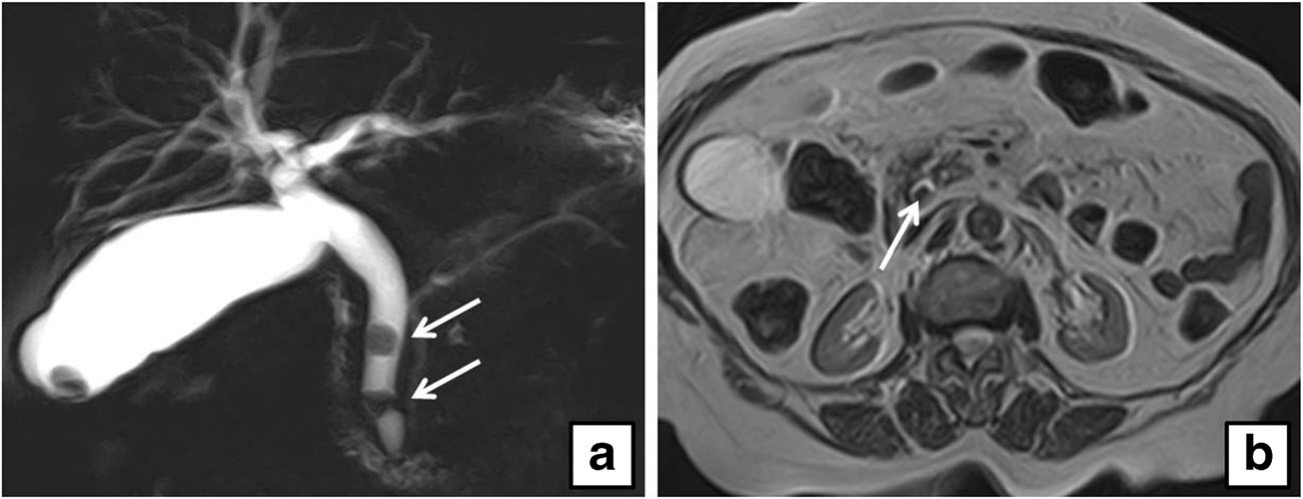
**a** Coronal maximum intensity projection (MIP) reformat shows two filling defects in a dilated CBD (*arrows*) and one in the gallbladder. **b** Axial T2-weighted MRI of the same patient shows a filling defect in a dilated CBD (*arrow*)

**Fig. 3:**
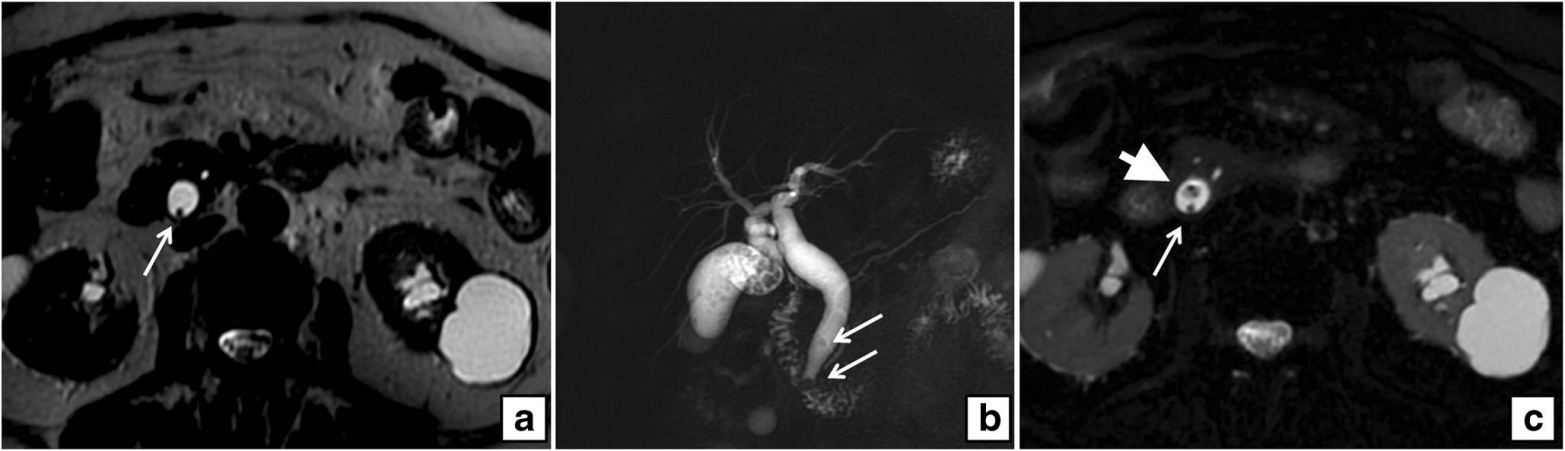
**a** Axial T2-weighted MRI shows a millimetric filling defect in a dilated CBD (*arrow*). **b** Coronal MIP reformat of the same patient shows multiple filling defects in the gallbladder and two stones are seen in the distal CBD (*arrows*) with mild upstream dilatation. **c** Axial fat-saturated T2-weighted MRI of the same patient shows two filling defects in a dilated CBD (*arrow* and *arrowhead*) at the same level of **a**

**Fig. 4:**
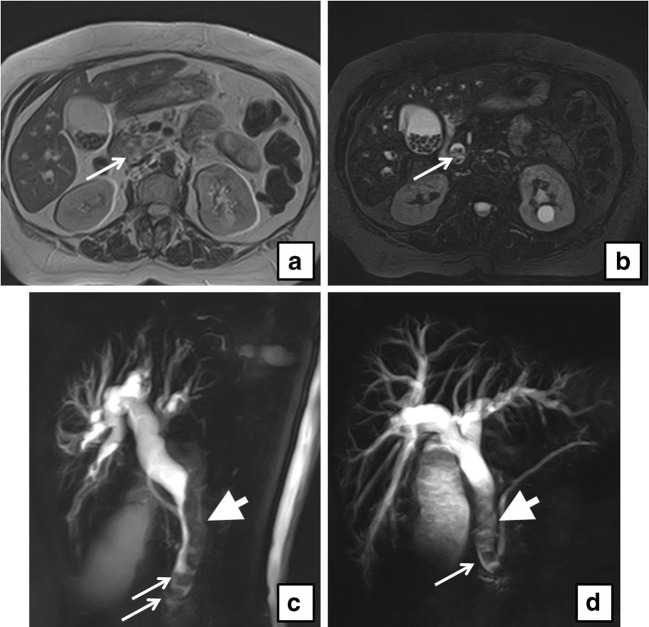
**a** Axial T2-weighted MRI shows multiple filling defects in the gallbladder and in the dilated CBD (*arrow*) . **b** Axial fat-saturated T2-weighted MRI of the same patient at the same level of **a**. **c**, **d** Coronal MIP reformat of the same patient shows multiple filling defects in the gallbladder and in the dilated CBD (*arrowhead*), and two stones are seen in the distal CBD (*arrows*)

On preoperative US examination, 5 out of 104 patients demonstrated a dilated CBD and, among them, only one patient (20%) presented CBD lithiasis on MRCP, whereas 6 out of 99 patients (6%) with normal choledocal diameter showed CBD lithiasis (Figs. 1, 2, 3, and 4).

With respect to the frequency of CBD lithiasis, MRCP showed calculi in 7 out of 104 patients with gallbladder stones awaiting cholecystectomy (6.7%), with no statistically significant differences between the groups with normal and altered AST/ALT (χ^2^ = 0.82; *p* = 0.36), ALP (χ^2^ = 0.05; *p* = 0.83), total serum bilirubin (χ^2^ = 0.05; *p* = 0.83) and choledocal diameter on preoperative US (χ^2^ = 1.47; *p* = 0.23).

The frequency of CBD lithiasis on MRCP and the relation with laboratory findings and choledocal diameter on preoperative US are presented in Table 2.

**Table 2:** Frequency of common bile duct (CBD) stones and relation with laboratory findings and choledocal diameter on preoperative ultrasound (US), chi-square (χ^2^) and *p*-value (*p*)

Laboratory and ultrasound findings	No. of patients	No. of patients with CBD stones	No. of patients without CBD stones	χ^2^; *p*
AST or ALT				
Normal AST or ALT	87	5 (5.7%)	82 (94.2%)	χ^2^ = 0.82; *p* = 0.36
Increased AST or ALT	17	2 (11.8%)	15 (88.2%)	
ALP				
Normal ALP	18	1 (5.5%)	17 (94.4%)	χ^2^ = 0.05; *p* = 0.83
Increased ALP	86	6 (7%)	80 (93%)	
Total bilirubin				
Normal bilirubin	86	6 (7%)	80 (93%)	χ^2^ = 0.05; *p* = 0.83
Increased bilirubin	18	1 (5.6%)	17 (94.4%)	
Choledocal diameter on ultrasound				
Normal (< 8 mm)	99	6 (6%)	93 (94%)	χ^2^ = 1.47; *p* = 0.23
Dilated (> 8 mm)	5	1 (20%)	4 (80%)	

The sensitivity and specificity of AST/ALT (PPV: 12%; NPV: 94%), ALP (PPV: 7%; NPV: 94%), total serum bilirubin (PPV: 6%; NPV: 93%) and choledocal diameter (PPV: 20%; NPV: 94%) were, respectively, 28.6 and 94.8%, 85.7 and 17.5%, 14.3 and 93.8%, and 14.3 and 95.9% (Table 3).

**Table 3:** Sensitivity, specificity, positive and negative predictive values (PPVs and NPVs, respectively) of laboratory findings and choledocal diameter on preoperative US

Laboratory and ultrasound findings	Sensitivity	Specificity	PPV	NPV
AST/ALT	28.6%	94.8%	12%	94%
ALP	85.7%	17.5%	7%	94%
Total serum bilirubin	14.3%	93.8%	6%	93%
Choledocal diameter	14.3%	95.9%	20%	94%

So the patients were classified into four different risk groups and the difference in frequency of CBD lithiasis was not statistically significant between the high/moderate risk and low/no risk groups (χ^2^ = 0.00; *p* = 0.95). The frequencies of CBD lithiasis in each group are presented in Table 4.

**Table 4:** Correlation between risk groups and frequency of CBD lithiasis on preoperative magnetic resonance cholangio-pancreatography (MRCP)

Risk group	No. of patients	No. of patients with CBD stones
High	21 (20.2%)	2 (1.9%)
Moderate	69 (66.3%)	4 (3.8%)
Low	9 (8.7%)	1(1%)
No risk	5 (4.8%)	0 (0%)
Total	104	7 (6.7%)

## Discussion

CBD stones are detected in 8–20% of patients undergoing cholecystectomy for cholelithiasis [9–11], of which 5% are asymptomatic [2].

Patients presenting with CBD stones may have symptoms including biliary colic, jaundice, cholangitis, pancreatitis or may be asymptomatic.

In our results, in line with previous studies, the incidence of CBD stones is 6.7%, 8.6% of which is asymptomatic.

Many studies have been performed to seek predictive tools for the presence of CBD stones in patients with gallbladder stones awaiting cholecystectomy, showing that preoperative laboratory findings and US evaluation of choledocal diameter were individually significant predictive factors and the likelihood of CBD stones increases up to 99% when all the predictors were positive [11].

Liver function tests can be used to screen for CBD stones, but these are neither highly sensitive nor specific [6, 7]. Elevated serum bilirubin and ALP typically reflect biliary obstruction, but serum bilirubin levels may be elevated or not according to the obstruction (complete or incomplete); ALP is usually associated with symptomatic CBD stones [12] and has the highest sensitivity in the diagnosis of choledocholithiasis among the biochemical parameters [13].

Videhult et al. [14] showed that almost half (48%) of the patients with CBD stones had normal ALP and bilirubin values. Thus, by using only findings of normal ALP and bilirubin as indicators of the absence of CBD stones would imply that half of CBD stones would remain undetected.

Similar conclusions were achieved by Abboud et al. [15] about bilirubin, ALP and amylase values, showing that positive likelihood ratios for dilated CBD on US, hyperbilirubinaemia and jaundice ranged from almost 4 to almost 7. Elevated levels of ALP and hyperamylasaemia exhibited positive likelihood ratios of less than 3.

Our data are in line with previous studies: the sensitivity of liver function tests is low overall, with the highest sensitivity achieved by serum ALP (85.7%).

Greater accuracy can be obtained by using laboratory findings in addition to imaging modalities [16, 17].

Non-invasive imaging techniques such as US are widely used for the diagnosis and monitoring of many biliary diseases. A CBD diameter greater than 6 mm on US is associated with a higher prevalence of choledocholithiasis [18].

However, these techniques have limitations, such as the low sensitivity of US for detecting CBD calculi, detecting only from 33 to 55% of the CBD stones [19]. In our results, the sensitivity of the US (14.3%) is clearly lower than other studies; this is explainable with a large variability in sensitivity due to the fact that US is an operator-dependent technique [20–23].

Computed tomography is associated with ionising radiation and is unreliable for detecting non-calcified stones.

ERCP was accepted as the ‘gold standard’ for bile duct imaging, with the advantage of permitting the bile duct to be cleared of stones. However, it is an invasive technique, with a reported mortality rate of 0.1–3% [11] and complications including pancreatitis, cholangitis, perforation and bleeding. Ideally, its use should be restricted to therapeutic procedures alone and not recommended as a routine examination. Intravenous cholangiography has the same sensitivity and specificity as ERCP [24]; however, it is an invasive technique and is associated with ionising radiation.

MRCP is a non-invasive imaging technique that does not require the use of X-rays or contrast media. The most important drawbacks of MRCP are its inability to offer therapeutic interventions and its high cost.

The criteria for a positive MRCP were signal defects within the CBD, defined variably as foci or rounded and oval in some studies [25].

MRCP has been shown to demonstrate normal and variant biliary anatomy accurately, as well as benign and malignant causes of bile duct obstruction with a sensitivity and specificity of 95–100% in the detection of CBD stones [19, 26].

In 2003, Romagnuolo et al. [27] reported an authoritative meta-analysis of 67 published controlled trials, showing that MRCP has an excellent overall sensitivity of 95% and a specificity of 97% for demonstrating CBD stones. Our results, with sensitivity and specificity of 100% for demonstrating CBD stones, are, therefore, in line with those previously reported.

Accuracy in detecting CBD stones, absence of ionising radiation and greater safety make MRCP a competitive diagnostic method in patients with biliary obstruction.

To our knowledge, there are different studies evaluating prospectively a scoring system designed to improve the accuracy of CBD stone prediction before laparoscopic cholecystectomy and to minimise the number of preoperative MRCP [28, 29].

However, we tried to use our data and insert them into a scoring system [8], showing that, among all patients undergoing cholecystectomy, bile duct stones would have been undetected in 8.6% due to the lack of symptoms and laboratory signs (patients in the no risk and low risk groups). These results are in line with previous similar studies in the literature [19, 30].

This strong concealment may lead to serious consequences for patients and associated overall healthcare costs [31].

This article does not analyse the cost-effectiveness for patients; however, based on this study, the importance of preoperative MRCP as the only non-invasive tool in screening patients with suspected choledocholithiasis was clearly demonstrated. MRCP can allow the surgeon to know the state of the patient’s biliary ductal condition and also the possible presence of severe inflammation that is reported as one of the most important reasons for bile duct injury [32].

For this reason, MRCP routine use, besides being diagnostically useful in the perioperative management in some cases, might be justified, despite its cost.

MRCP is a reliable and non-invasive evaluation for the detection or exclusion of CBD stones, reducing the misdiagnosis of retained choledocholithiasis with normal biochemical predictors and normal US examination. Thus, routine preoperative MRCP examination is suggested in patients who are candidates for cholecystectomy because of a gallstone disease.
